# Application of the Lancet Commission Criteria for the Diagnosis of Obesity to a Clinical Trials Population: The LEAP Trial

**DOI:** 10.1002/oby.70070

**Published:** 2025-10-27

**Authors:** Faith Anne N. Heeren, Kathleen R. Ruddiman, Courtney Simmons, Brian N. White, Byron C. Jaeger, Nicholas M. Pajewski, Stephanie A. Hooker, Deborah B. Horn, Katy Martin‐Fernandez, Kimberly A. Gudzune, Caroline Blackwell Young, Jamy Ard, Kristina Henderson Lewis

**Affiliations:** ^1^ Department of Implementation Science Wake Forest University School of Medicine Winston‐Salem North Carolina USA; ^2^ Department of Internal Medicine, Endocrinology Section Wake Forest University School of Medicine Winston‐Salem North Carolina USA; ^3^ Department of Biostatistics and Data Science Wake Forest University School of Medicine Winston‐Salem North Carolina USA; ^4^ HealthPartners Institute Minneapolis Minnesota USA; ^5^ Department of Family Medicine and Community Health University of Minnesota Minneapolis Minnesota USA; ^6^ Department of Surgery University of Texas McGovern Medical School Houston Texas USA; ^7^ Atrium Health Wake Forest Baptist, Weight Management Center Winston‐Salem North Carolina USA; ^8^ Department of Surgery Wake Forest University School of Medicine Winston‐Salem North Carolina USA; ^9^ American Board of Obesity Medicine Foundation Denver Colorado USA; ^10^ Department of Epidemiology and Prevention Wake Forest University School of Medicine Winston‐Salem North Carolina USA

**Keywords:** clinical care, diagnosis, obesity medication, obesity treatment, pharmacotherapy

## Abstract

**Objective:**

This study aimed to apply *The Lancet Diabetes & Endocrinology* criteria for diagnosing obesity among clinical trial participants and understand participants' characteristics by obesity status.

**Methods:**

The criteria were operationalized and applied to baseline data from the Long‐term Effectiveness of the Anti‐obesity medication Phentermine (LEAP) trial (NCT05176626). Excess adiposity, organ and tissue dysfunction, and limitations to daily activities were assessed. We examined differences between participants with “no obesity,” “pre‐clinical obesity,” and “clinical obesity.”

**Results:**

Among the 860 participants, 0.8% had no obesity (mean BMI 29.0 kg/m^2^ [SD 0.6]), 18.7% had pre‐clinical obesity (mean BMI 35.2 kg/m^2^ [SD 3.5]), and 80.5% had clinical obesity (mean BMI 35.9 kg/m^2^ [SD 4.3]). Participants with no/pre‐clinical obesity had lower mean SF‐12 mental component scores and greater baseline engagement with weight control strategies compared to those with clinical obesity. Participants with clinical obesity were older and, by definition, had a greater burden of cardiometabolic risk factors.

**Conclusions:**

Among clinical trial participants eligible for obesity pharmacotherapy, 19.5% were classified as having no/pre‐clinical obesity using the Lancet criteria. Applying the criteria was complicated in a well‐resourced trial setting, which suggests potential challenges in implementing these guidelines in real‐world practice.

**Study Importance**

- What is already known?
  ○ In 2025, *The Lancet Diabetes & Endocrinology* published a commission report detailing recommended criteria for the diagnosis of obesity. The implications of applying these criteria in clinical and research settings are unclear.
- What does this study add?
  ○ This study attempted to apply the Lancet Commission criteria to baseline data for an ongoing clinical trial of obesity pharmacotherapy. Patients in the “pre‐clinical obesity” group were younger and more likely to be female but had many similarities to those with “clinical obesity.” Thus, additional guidance on the clinical application of the proposed criteria is needed.
- How might these results change the direction of research or clinical practice?
  ○ All participants in the clinical trial met Food and Drug Administration (FDA) indications for pharmacological treatment with obesity medication. However, according to the Lancet Commission criteria, those with pre‐clinical obesity may be offered primarily lifestyle intervention unless they are deemed high risk. Further guidance on determination of high risk of disease progression is needed.

## 1 Introduction

In 2025, a comprehensive set of new criteria for the diagnosis of obesity was proposed in *The Lancet Diabetes & Endocrinology* [1]. Briefly, the criteria recommend first assessing and confirming excess adiposity, then determining whether dysfunction of tissue or organ systems is present and/or if patients have age‐adjusted limitations to daily activities. Depending on the findings of these assessments, patients may be classified as having “no obesity,” “pre‐clinical obesity,” or “clinical obesity” [1].

The proposed criteria define obesity by the presence of organ or tissue dysfunction in addition to excess adiposity. Although the report has been widely endorsed, the impact of the proposed criteria in clinical and research settings is unknown, as is their benefit above simply measuring body mass index (BMI) [2]. The present study operationalized the Lancet criteria using baseline data among participants in an ongoing clinical trial.

## 2 Methods

The current study is a secondary analysis of baseline data from the LEAP (Long‐term Effectiveness of the Anti‐obesity medication Phentermine) trial, an ongoing 24‐month, double‐blinded study examining weight loss and change in systolic blood pressure among adults treated with phentermine versus placebo. A comprehensive description of the LEAP trial can be viewed on ClinicalTrials.gov (NCT05176626). Full inclusion and exclusion criteria are listed in Table S1. The Wake Forest University School of Medicine's Institutional Review Board approved the study (IRB00079100).

To operationalize the Lancet criteria among LEAP participants, a logic flow was applied to baseline data (Table S2). First, excess adiposity was assessed and confirmed using specified cutoffs for BMI, waist circumference (WC) [3], and waist to height ratio (WtHR) measurements based on race, ethnicity, and sex.

Among participants meeting criteria for excess adiposity, tissue and organ dysfunction and limitations to daily activities were assessed using self‐report, diagnostic, or survey measures from the LEAP trial database. Self‐report of qualifying diagnoses was collected by study clinicians during the run‐in period for the trial. Diagnostic tests included blood pressure [4] and fasting bloodwork. For some criteria, clinicians trained in obesity medicine (K.R.R. and K.H.L.) determined appropriate values that would indicate criteria were met. Survey measures included assessment of limitations to daily activities through the Short Form Health Survey (SF‐12) physical functioning scores, with scores < 50 indicating limitations to daily activities [5]. Additional behavioral self‐report surveys were used, including the Weight Control Strategies Scale (WCSS), the short‐form Weight Efficacy Lifestyle Questionnaire (WEL‐SF), and the International Physical Activity Questionnaire (IPAQ) [6, 7, 8].

After assessment, participants were categorized as having: (1) no obesity (excess adiposity not confirmed), (2) pre‐clinical obesity (excess adiposity confirmed, tissue and organ function are preserved, no limitations to daily activities), or (3) clinical obesity (excess adiposity confirmed, tissue and organ dysfunction and/or limitations to daily activities are present). We compared groups to identify clinical and demographic differences. Because of the small cell sizes introduced by the no obesity group, statistical differences between the groups on measured characteristics were evaluated using either the Kruskal‐Wallis rank sum or Fisher's exact test.

## 3 Results

The logic flow was applied to 870 randomized trial participants. A small number (*n* = 10) were missing demographic and/or anthropometric data and were excluded. Seven participants (0.8% of those evaluated) did not meet criteria for excess adiposity and thus were categorized as having no obesity. Among this group, none qualified as having obesity based on BMI criteria alone, but all were eligible to participate in the LEAP trial due to the presence of a weight‐related comorbidity. Of all trial participants evaluated, 18.7% (*n* = 161) were characterized as having pre‐clinical obesity and 80.4% (*n* = 692) as having clinical obesity.

Demographic and clinical characteristics of these three groups are summarized in Tables 1 and 2 and Table S3. Participants defined as having no obesity had lower mean (SD) BMI (29.0 [0.6]), while the BMI of those in the pre‐clinical obesity (35.2 [3.5]) and clinical obesity (35.9 [4.3]) groups was very similar. Those with pre‐clinical obesity were significantly younger (43.2 years [11.9]) compared to those with clinical obesity (50.1 years [11.8]). The burden and severity of cardiometabolic disease and comorbidities were, by definition, higher among participants categorized as having clinical obesity; however, the clinical and population health significance of these differences was small (e.g., 30.5% of those with clinical obesity had prediabetes versus 24.2% among those with pre‐clinical obesity and 28.6% among those with no obesity; Table 2).

**TABLE 1:** Differences in demographic variables between trial participants characterized as having “clinical obesity” versus “pre‐clinical obesity” versus “no obesity” using Lancet Commission criteria.

Characteristic	Overall (*N* = 860)^a^	Clinical obesity (*N* = 692)^a^	Pre‐clinical obesity (*N* = 161)^a^	No obesity (*N* = 7)^a^	*p* ^b^
Age, years	48.8 (12.1)	50.1 (11.8)	43.2 (11.9)	53.0 (8.7)	< 0.001
Sex at birth					< 0.001
Male	234 (27.2%)	203 (29.3%)	27 (16.8%)	4 (57.1%)	
Female	626 (72.8%)	489 (70.7%)	134 (83.2%)	3 (42.9%)	
Race/ethnicity					0.8
Non‐Hispanic White	521 (60.8%)	424 (61.4%)	91 (56.9%)	6 (85.7%)	
Non‐Hispanic Black	151 (17.6%)	121 (17.5%)	29 (18.1%)	1 (14.3%)	
Hispanic	146 (17.0%)	113 (16.4%)	33 (20.6%)	0 (0.0%)	
Other	39 (4.6%)	32 (4.6%)	7 (4.4%)	0 (0.0%)	
Unknown	3	2	1	0	
Education					0.2
High school or less	81 (9.5%)	72 (10.5%)	8 (5.0%)	1 (16.7%)	
Some post‐high school education	271 (31.9%)	219 (32.0%)	50 (31.4%)	2 (33.3%)	
College graduate or more	497 (58.5%)	393 (57.5%)	101 (63.5%)	3 (50.0%)	
Unknown	11	8	2	1	

^a^ Mean (SD); *n* (%).

^b^ Kruskal–Wallis rank sum test; Fisher's exact test.

**TABLE 2:** Differences in clinical and cardiometabolic health measures between trial participants characterized as having “clinical obesity” versus “pre‐clinical obesity” versus “no obesity” using Lancet Commission criteria.

Characteristic	Overall (*N* = 860)^a^	Clinical obesity (*N* = 692)^a^	Pre‐clinical obesity (*N* = 161)^a^	No obesity (*N* = 7)^a^	*p* ^b^
BMI, kg/m^2^	35.7 (4.2)	35.9 (4.3)	35.2 (3.5)	29.0 (0.6)	< 0.001
BMI, kg/m^2^ categorizations					< 0.001
25 to < 30: Overweight	54 (6.3%)	39 (5.6%)	8 (5.0%)	7 (100.0%)	
30 to < 35: Obesity Class I	350 (40.7%)	274 (39.6%)	76 (47.2%)	0 (0.0%)	
35 to < 40: Obesity Class II	292 (34.0%)	233 (33.7%)	59 (36.6%)	0 (0.0%)	
40+: Obesity Class III	164 (19.1%)	146 (21.1%)	18 (11.2%)	0 (0.0%)	
Waist circumference, cm	113.3 (11.8)	114.3 (11.6)	109.9 (9.6)	87.5 (22.9)	< 0.001
Systolic blood pressure, mm Hg	120.0 (11.8)	121.6 (11.7)	112.8 (9.4)	123.1 (7.1)	< 0.001
Diastolic blood pressure, mm Hg	80.5 (7.8)	81.6 (7.8)	75.4 (6.1)	81.7 (7.4)	< 0.001
HbA1c, %	5.7 (0.6)	5.8 (0.6)	5.6 (0.5)	5.7 (0.7)	< 0.001
Unknown	1	0	1	0	
Triglycerides, mg/dL	125.9 (64.3)	130.4 (67.0)	107.3 (48.5)	101.0 (27.7)	< 0.001
Unknown	1	0	1	0	
LDL, mg/dL	119.1 (31.6)	119.6 (31.7)	117.4 (30.4)	100.6 (43.7)	0.2
Unknown	1	0	1	0	
HDL, mg/dL	52.2 (13.6)	51.8 (13.7)	53.9 (12.7)	53.1 (21.1)	0.11
Unknown	1	0	1	0	
10‐year estimated ASCVD risk, %	5.1 (5.1)	5.5 (5.3)	2.6 (3.3)	3.6 (2.3)	< 0.001
Unknown	260	182	76	2	
Prediabetes	251 (29.3%)	210 (30.5%)	39 (24.2%)	2 (28.6%)	0.3
Unknown	4	4	0	0	
Type 2 diabetes mellitus	56 (6.5%)	51 (7.4%)	4 (2.5%)	1 (14.3%)	0.025
Unknown	1	1	0	0	
Dyslipidemia	437 (51.2%)	371 (54.1%)	62 (38.8%)	4 (57.1%)	0.001
Unknown	7	6	1	0	
Depression	112 (13.1%)	96 (13.9%)	16 (10.1%)	0 (0.0%)	0.3
Unknown	3	1	2	0	
Anxiety	154 (17.9%)	116 (16.8%)	35 (21.9%)	3 (42.9%)	0.061
Unknown	2	1	1	0	

^a^ Mean (SD); *n* (%).

^b^ Kruskal–Wallis rank sum test; Fisher's exact test.

Self‐reported behavioral and quality of life characteristics are summarized across groups in Table 3. All groups exhibited similar levels of self‐reported physical activity, but the pre‐clinical obesity and no obesity groups had higher mean scores on the WCSS compared to those with clinical obesity. Additionally, those with no obesity and pre‐clinical obesity scored lower on the mental component of the SF‐12.

**TABLE 3:** Differences in lifestyle behavior variables between trial participants characterized as having “clinical obesity” vs. “pre‐clinical obesity” vs. “no obesity” using Lancet Commission criteria.

Characteristic	Overall (*N* = 860)		Clinical obesity (*N* = 692)		Pre‐clinical obesity (*N* = 161)		No obesity (*N* = 7)		
	*N*	Mean (SD)	*N*	Mean (SD)	*N*	Mean (SD)	*N*	Mean (SD)	*p* ^f^
WCSS^a^—total average score	847	1.4 (0.6)	685	1.4 (0.6)	155	1.5 (0.6)	7	1.6 (0.6)	0.002
IPAQ^b^—total MET minutes of physical activity per week	823	2291.6 (2711.8)	663	2275.9 (2664.9)	153	2255.7 (2619.7)	7	4570.5 (6632.6)	0.5
WEL‐SF^c^—total score	836	49.4 (17.9)	675	49.1 (18.0)	154	50.9 (17.7)	7	38.1 (8.5)	0.092
SF‐12^d^—physical component	812	47.9 (7.2)	663	46.8 (7.4)	142	53.4 (2.4)	7	49.0 (6.1)	< 0.001
SF‐12^d^—mental component	812	51.7 (7.8)	663	52.1 (7.6)	142	49.8 (8.0)	7	48.4 (9.2)	0.002
SF‐12^d^, ^e^—physical component < 50	812	405 (49.9%)	663	402 (60.6%)	142	0 (0.0%)	7	3 (42.9%)	< 0.001
SF‐12^d^—mental component < 50	812	263 (32.4%)	663	205 (30.9%)	142	55 (38.7%)	7	3 (42.9%)	0.15

^a^ WCSS = Weight Control Strategies Scale, assessing recent self‐reported engagement in dietary, exercise, and other behaviors known to reduce weight. WCSS scores range from 0 to 4, with a higher score indicative of greater engagement in weight‐controlling behaviors.

^b^ IPAQ = International Physical Activity Questionnaire, assessing self‐reported moderate and vigorous physical activity over the past week in terms of METs (metabolic equivalents). At a minimum, the Department of Health and Human Services recommends at least 150 min of moderate to vigorous physical activity per week, which is equivalent to approximately 500 MET minutes per week.

^c^ WEL‐SF = Weight Efficacy Lifestyle Questionnaire, Short Form, which measures self‐reported confidence in the ability to resist overeating, with scores ranging from 0 to 80 points, where higher scores correspond to greater confidence levels.

^d^ SF‐12 = Short Form Health Survey is a 12‐item measure of quality of life with domains focused on both physical and mental health. The SF‐12 includes two summary scores: the physical component summary and the mental component summary. For each summary, score < 50 is indicative of below average health.

^e^ By definition, participants in the pre‐clinical obesity category have a SF‐12 physical component score ≥ 50.

^f^ *p* value based on Kruskal–Wallis rank sum test and Fisher's exact test.

## 4 Discussion

The present study applied the 2025 Lancet Commission criteria for the diagnosis of obesity to baseline data from an ongoing clinical trial of long‐term phentermine monotherapy. All participants met current Food and Drug Administration (FDA) indications for pharmacological treatment, yet approximately one in five were grouped as having “no obesity” or “pre‐clinical obesity” using the Lancet Commission categorization; thus, they may not qualify for pharmacotherapy if the criteria were operationalized. Although several differences were noted, the groups differed primarily in characteristics that were inherent to the definition of clinical obesity (e.g., higher rates of obesity comorbidities such as prediabetes and diabetes in those with “clinical obesity”). Importantly, the criteria proved complex to operationalize, even in the context of a rich clinical trial dataset.

Patients with clinical obesity in our sample had more cardiometabolic risk factors. This pattern demonstrates that the application of the Lancet Commission criteria does identify patients where excess adiposity affects health status. However, many in the pre‐clinical obesity group would currently be considered to have higher severity obesity: 36.6% of this group have Class II obesity (BMI 35.0–39.9) and 11.2% have Class III obesity (BMI ≥ 40). Notably, the age difference between groups was one of the largest clinical differences observed. This pattern may reflect disease progression over time as older adults with obesity have a higher risk of hypertension, chronic kidney disease, and diabetes [9, 10, 11].

Considering the discrepancy between FDA treatment cut points and treatment recommendations based on the Lancet Commission categories, more information is needed on the impact of initial treatment choice for those categorized as having pre‐clinical or no obesity. The Lancet Commission report recommends lifestyle intervention and continued monitoring for patients with pre‐clinical obesity, unless a clinician determines the patient is at high risk of disease progression [1]. Making these determinations on an individual level may be problematic in practice as clinician bias has been shown to impact the quality of clinical care, potentially reducing obesity medication prescriptions and referral for surgical intervention [12, 13, 14]. Subsequently, the implications of the application of the proposed criteria by insurance companies for approval of treatment options are unclear. Importantly, all participants in the LEAP trial expressed a desire to lose weight and had a sufficiently high disease burden to enroll in a lengthy, placebo‐controlled trial. Thus, clearer recommendations for diagnostic indications, policy implications, and assessment of patients' perceived disease burden are needed to inform treatment choices.

By leveraging a rich clinical trial dataset, this study provides preliminary data on psychosocial measures not routinely assessed. The Lancet Commission report recognizes the impact of stigma on patients; however, psychological health constructs are absent from the criteria given lack of clear evidence. The relationship between obesity and psychological health is complex, but obesity and psychiatric disorders often co‐occur [15, 16, 17]. In this sample, those with pre‐clinical obesity had poorer mental quality of life scores when compared to those with clinical obesity, potentially indicating psychological distress [18]. Application of the criteria in a broader context with consideration of psychological health is needed to understand its role in the diagnostic framework.

This study was limited by our inability to assess all 18 organ system and/or tissue dysfunctions specified in the Lancet Commission report. We relied on self‐report and clinical judgment for several criteria (Table S2), as it was not possible to access the full medical records of all trial participants. In comparison to the Lancet Commission criteria, the Department of Veterans Affairs guidelines recommend assessment for 9 common obesity‐associated conditions in the primary care setting; thus, real‐world assessment for 18 areas of organ dysfunction using the Lancet Commission criteria may prove more costly and time‐intensive than current clinical practice recommendations [19]. Another limitation was the predominantly non‐Hispanic White and female population with higher educational attainment, which potentially limits generalizability. Lastly, the current study, like other recent analyses applying the proposed criteria, is cross‐sectional [2, 19]. Longitudinal analyses are needed to investigate how the proposed criteria track disease progression and remission, particularly for those in each diagnostic group who receive evidence‐based treatments.

Given the limitations of the LEAP dataset, it is possible the prevalence of clinical obesity in the current sample is overestimated. For example, since the publication of the Lancet report, it has been clarified that the waist to hip ratio should be used in addition to waist circumference to confirm excess adiposity [20]. Unfortunately, we were unable to use waist to hip ratios as hip circumference was not measured on trial participants. Additionally, it has been clarified that an identified system, tissue, and/or organ dysfunction must be directly attributed to excess adiposity in order for it to confirm a clinical obesity diagnosis [20]. Because some portion of the population may have that dysfunction for reasons other than excess adiposity (e.g., essential hypertension in the absence of excess adiposity), counting all adults with obesity as having these complications due to excess adiposity may overestimate the true prevalence of clinical obesity (and, in turn, underestimate the prevalence of pre‐clinical obesity). For example, in data from the National Health and Nutrition Examination Survey, 15.6% of adults in the United States with hypertension had a normal BMI [21]. In our sample, among those with clinical obesity, 72.4% had hypertension. It is possible that some subset of patients in the clinical obesity group may have had hypertension independent of their weight status. However, due to the cross‐sectional nature of our analysis, we are unable to determine whether each comorbidity was directly attributable to excess adiposity for an individual patient. Future studies should consider complementary anthropometric measures and collect prospective data or develop other methods to systematically determine whether comorbidities may be directly attributed to excess adiposity.

## 5 Conclusion

The present study applied the Lancet Commission criteria to baseline data from a clinical trial for pharmacological treatment. Some differences between groups were noted, but the clinical meaningfulness of these differences and implications for treatment warrant further investigation. Additionally, policy implications (e.g., insurance coverage) of the proposed criteria should also be determined. Future studies should longitudinally assess the Lancet Commission categorization and the effects of treatment choice among samples diverse in race, ethnicity, sex, and education.

## Conflicts of Interest

F.A.N.H. reports serving on The Obesity Society's Finance Committee and a leadership position within the Obesity and Eating Disorders Special Interest group associated with the Society of Behavioral Medicine. She also reports membership on the Obesity Action Coalition's Access to Care and Membership committees, consulting fees from WW International Inc., and an honorarium for speaking from the American Academy of Pediatrics. D.B.H. reports consulting fees from Amgen, AstraZeneca, Boehringer Ingelheim, Eli Lilly and Company, Novo Nordisk, and Zealand. D.B.H. reports institutional research support from Eli Lilly and Company and Novo Nordisk. D.B.H. reports serving on the World Obesity Federation and the Program Committee for The Obesity Society. K.A.G. is employed by the American Board of Obesity Medicine Foundation and was previously employed by the American Board of Obesity Medicine. She has received personal fees as a conference speaker from the American College of Cardiology, the American Diabetes Association, and PRI‐MED; personal fees for participation on advisory boards for Eli Lilly and Company and Novo Nordisk; and travel support from the American College of Cardiology, the American Diabetes Association, Eli Lilly and Company, and Novo Nordisk. She has received royalties from Johns Hopkins ACG System. Her former institution (Johns Hopkins) received grant funding from Novo Nordisk. J.A. reports grants or contracts from the following organizations: Nestle Healthcare Nutrition, Eli Lilly and Company, Boehringer Ingelheim, Epitomee Inc., UnitedHealth Group R&D, KVKTech, WW International Inc., and Regeneron. J.A. also reports consulting fees from Nestle Healthcare Nutrition, Eli Lilly and Company, Optum Labs R&D, Novo Nordisk, Intuitive, Regeneron, Brightseed, WW International Inc., Amgen, and Boehringer Ingelheim. J.A. reports leadership or fiduciary roles on The Obesity Society (President) and the American Society for Nutrition Foundation (Executive Board Member) and receipt of equipment, materials, drugs, or other gifts from KVK Tech Pharmaceuticals, WW International Inc., and Nestle Healthcare Nutrition. K.H.L. reports being a sub‐investigator for a trial funded by Amgen, payments from the American Board of Obesity Medicine for participation in meetings, payments from Novo Nordisk for participation on an advisory board, an honorarium from Vindico Medical Education for a CME event, and an honorarium for an invited lecture from the American Diabetes Association. K.H.L. also reports support from Novo Nordisk, Amgen, and the American Board of Obesity Medicine for travel and meetings and membership on the American Board of Obesity Medicine's Board of Directors. The other authors declared no conflicts of interest.

## Supporting information


**Table S1:** Full inclusion and exclusion criteria for the Long‐term effectiveness of the anti‐obesity medication phentermine (LEAP) trial.
**Table S2:** Logic flow operationalizing Lancet Commission Criteria for application to baseline data.
**Table S3:** Description of participants characterized as having “clinical obesity.”

## Data Availability

The data used in this report are from an ongoing clinical trial. Data will be made available in a public repository at the time of the primary results publication consistent with NIH policies.
