# Supplementary material for: Application of the Lancet Commission Criteria for the Diagnosis of Obesity to a Clinical Trials Population: The LEAP Trial
Source: Obesity (Silver Spring). 2025 Oct 27;34(1):58–64. doi: 10.1002/oby.70070 (PMC12724050; doi:10.1002/oby.70070)
Supplement: Supplementary file 1 — Table S1: Full inclusion and exclusion criteria for the Long‐term effectiveness of the anti‐obesity medication phentermine (LEAP) trial. Table S2: Logic flow operationalizing Lancet Commission Criteria for application to baseline data. Table S3: Description of participants characterized as having “clinical obesity.” [file OBY-34-58-s001.docx]

**Supplementary Table 1: Full inclusion and exclusion criteria for the Long-term effectiveness of the anti-obesity medication phentermine (LEAP) trial**

| Inclusion Criteria | - Aged 18-70 - Body mass index 30-44.9 kg/m^2^ OR BMI 27-29.9 kg/m^2^ with weight-related comorbidity - English-speaking - Have an internet-connected device - Interested in losing weight - Able to adhere to an oral medication - Able to adhere to required study visits - For females of reproductive potential: use effective contraception during study period - Willingness to apply to study procedures |
| --- | --- |
| Exclusion Criteria | - History of coronary artery, cerebrovascular, or peripheral arterial disease - Poorly controlled blood pressure or elevated heart rate - History of cardiac arrythmia - Active or currently treated hyperthyroidism - Poorly controlled or undertreated hypothyroidism - History of glaucoma or indicated high risk of glaucoma - Heavy alcohol use within the last 6 months - History of substance use disorder or use of illicit substances in the past 12 months - Use of a drug in the monoamine oxidase inhibitor class within the last 14 days - Currently pregnant or breastfeeding; trying to conceive; seeking or in active treatment for infertility - End-stage renal disease on dialysis or CKD class IV or higher - History of valvular heart disease - Congestive heart failure - Cirrhosis or symptoms of liver failure - Severe pulmonary disease requiring supplemental oxygen - Cessation of nicotine-containing products <6 months prior to baseline - Use of oral corticosteroids more than 5 days in the last 3 months - Elevated depressive symptoms - Uncontrolled anxiety symptoms - Hospitalization for mental illness in the last 24 months - Diagnosis of dementia or serious mental illness - Eating disorder diagnosis or treatment within the past 2 years - Prior procedure for weight control - Use of anti-obesity medication with similar mechanism in the past 24 months - Use of any anti-obesity medication in the past 12 months - Unstable dose of weight loss promoting medications in the last 12 months - Use of any stimulant medications in the past month - Known allergy or intolerance to phentermine - Documented/self-reported weight change of >5% in past 3 months - History of cancer (other than non-melanoma skin cancer) in the past 5 years - History of organ transplantation - Body weight >400 pounds - Upper arm circumference exceeds 50 centimeters - Plan to move outside the area in the next two years - Unable to make changes to diet - Participation in a similar study - Other concerns/comorbidities that may prevent participant from taking part in the intervention |

**Supplementary Table 2: Logic flow operationalizing Lancet Commission Criteria for application to baseline data**

| **Step 1: Assess and confirm excess adiposity** | |
| --- | --- |
| Baseline variables utilized: | Consider the following based on race, ethnicity, and sex   - Body mass index (BMI) - Waist circumference (WC) - Waist-to-height ratio (WtHR)   BMI was first assessed based on race/ethnicity   - Asian participants:   - If BMI <27.5= assess WtHR and WC (both must be met to confirm excess adiposity). WtHR >0.50= excess adiposity. For women, WC >80 centimeters= confirmed excess adiposity. For men >85= confirmed excess adiposity.   - If BMI >27.5= assess WtHR and WC using the above cut-offs (only one criterion needs to be met) - Hispanic participants:   - If BMI <30, consider WtHR and WC (both criteria must be met). WtHR >0.50= excess adiposity. For women, WC of >80 cm= excess adiposity. For men, WC of >90= excess adiposity.   - If BMI >30, <40, consider WtHR and WC using the above cut-offs (only one criterion must be met)   - If BMI >40, excess adiposity is confirmed. - All other race/ethnicities, assess BMI   - If BMI <30, consider WtHR and WC (both criteria must be met). WtHR >0.50= excess adiposity. For women, WC of >88 cm= excess adiposity. For men, WC of >102= excess adiposity.   - If BMI >30, <40, consider WtHR and WC using the above cut-offs (only one criterion must be met)   - If BMI >40, excess adiposity is confirmed. |
| **Step 2: Assess evidence of reduced tissue/organ function and/or limitations on daily activities** | |
| Organ/tissue system | Baseline variables utilized |
| Upper airways | - Self-report of obstructive sleep apnea |
| Reproductive (Female) | - Self-report of polycystic-ovarian syndrome |
| Limitations of daily activities | - Short-form 12 (SF-12) physical functioning scores |
| Raised arterial pressure | - Self-report of hypertension - Documentation of hypertension with or without meds - Listed hypertensive medications at baseline - Baseline and/or randomization blood pressure measurement   - If SBP GE 140 at EITHER baseline or randomization= clinical obesity   - If DBP GE 90 at EITHER baseline or randomization= clinical obesity   - If SBP 131-139 at BOTH baseline and randomization= clinical obesity   - If DBP 80-89 at BOTH baseline and randomization= clinical obesity |
| Liver function | - Self-report of metabolic dysfunction-associated fatty liver disease (non-alcoholic fatty liver disease) - Assess age-adjusted cutoffs for FIB-4 scores using complete blood count, comprehensive metabolic panel, and waist circumference   - >35 and <65 years; FIB-4 >1.3 and <2.67= pre-clinical obesity; >2.67= clinical obesity   - >65 years; FIB-4 >2.0 and <2.67= pre-clinical obesity; >2.67= clinical obesity |
| Metabolism (cluster of all 3 must be present) | - A1C (>5.7) or fasting blood glucose >100mg/dL - Triglycerides (>150 mg/dl) - HDL cholesterol (For men, <40 mg/dL. For women <50, mg/dL) |
| **Step 3: If none of the above criteria are met, assess the patient for the following as documented in their baseline problem list or history and physical:** | |
| Baseline variables utilized: | - Problem list and/or History/Physical as recorded by study clinician scanned for the following diagnoses:   - Recurrent/chronic urinary incontinence   - Recurrent DV and/or pulmonary thromboembolic disease   - Hypogonadism   - Intracranial pressure or idiopathic intracranial hypertension   - Osteoarthritis^1^   - Peripheral artery disease |

*Based on trial design, participants did not meet criteria for some aspects of cardiovascular health due to exclusion criteria of the trial, thus these results may not fully generalize to other pharmacological trials with differing inclusion criteria. Despite robust data collection, we were unable to assess the respiratory, renal, and lymphatic systems using baseline trial data.

^1^The Lancet Commission report suggests assessing severe knee or hip pain with stiffness and reduced range of joint motion

**Supplementary Table 3: Description of Participants Characterized as having “Clinical Obesity”**

| **Characteristic** | **N = 692**^1^ |
| --- | --- |
| Excess adiposity confirmed |  |
| Yes | 692 (100.0%) |
| Number of criteria for clinical obesity met |  |
| 1 | 310 (44.8%) |
| 2 | 254 (36.7%) |
| 3+ | 128 (18.5%) |
| Organ Dysfunction vs Limitations of Activity Criteria Met |  |
| Both | 308 (44.5%) |
| Limitations of Activities Only | 94 (13.6%) |
| Organ Function Only | 290 (41.9%) |
| Self-report of obstructive sleep apnea |  |
| Yes | 125 (18.1%) |
| No | 566 (81.9%) |
| Unknown | 1 |
| Self-report of PCOS (Female only) |  |
| Yes | 29 (4.2%) |
| No | 662 (95.8%) |
| Unknown | 1 |
| SF-12 Physical component < 50 |  |
| Yes | 402 (60.6%) |
| No | 261 (39.4%) |
| Unknown | 29 |
| Raised arterial pressure |  |
| Yes | 501 (72.4%) |
| No | 191 (27.6%) |
| Self-report of NAFLD |  |
| Yes | 29 (4.2%) |
| No | 662 (95.8%) |
| Unknown | 1 |
| FIB-4 criteria met for clinical obesity |  |
| Yes | 2 (0.3%) |
| No | 689 (99.7%) |
| Unknown | 1 |
| Hyperglycemia, high triglyceride levels, and low HDL are present |  |
| Yes | 77 (11.1%) |
| No | 615 (88.9%) |
| Criteria met on medical history form* |  |
| Yes | 64 (9.3%) |
| No | 627 (90.7%) |
| Unknown | 1 |
| ^1^n (%) | |
| *Urinary incontinence, DVT and/or pulmonary thromboembolic disease, hypogonadism, intracranial pressure, idiopathic intracranial hypertension, osteoarthritis, peripheral artery disease | |
